# Conjunctival leiomyosarcoma in a patient with xeroderma pigmentosum: 5-year follow-up without recurrence

**DOI:** 10.5935/0004-2749.2021-0301

**Published:** 2022-07-04

**Authors:** Zeynep Şerikoğlu Akbaş, Bilge Batu Oto, Busenur Gönen, Övgü Aydın Ülgen, Ahmet Murat Sarıcı

**Affiliations:** 1. Department of Ophthalmology, Cerrahpasa Medical Faculty, Istanbul University- Cerrahpaşa, Istanbul, Turkey; 2. Department of Pathology, Cerrahpasa Medical Faculty, Istanbul University- Cerrahpaşa, Istanbul, Turkey

**Keywords:** Conjunctival neoplasms, Leiomyosarcoma, Xeroderma pigmentosum, Humans, Case report, Neoplasias da túnica conjuntiva, Leiomiossarcoma, Xeroderma pigmentoso, Humanos, Relato de caso

## Abstract

Conjunctival leiomyosarcoma is a very rare soft tissue malignancy. Herein, we describe a conjunctival leiomyosarcoma case in a patient with another rare disease, xeroderma pigmentosum. The 27-year-old single-eyed xeroderma pigmentosum patient complained of exophytic mass covering the ocular surface in her left eye. A vascular, hemorrhagic mass covering the entire ocular surface of the left eye was identified on the examination. Thus, total mass excision surgery was performed. The pathological diagnosis was compatible with conjunctival leiomyosarcoma. Additional chemotherapy, radiotherapy, or surgery were not accepted by the patient. No recurrence or metastasis was observed during the 5-year follow-up. Both primary conjunctival leiomyosarcoma and xeroderma pigmentosum are very rare diseases. Conjunctival masses in xeroderma pigmentosum patients should be approached carefully, and histopathological examination is warranted. For conjunctival leiomyosarcoma, early diagnosis, localized, unspread disease, and complete resection provide the best prognosis.

## Abstract (pt)

O leiomiossarcoma da conjuntiva é um tumor ma­ligno de tecidos moles muito raro. Aqui é descrito um caso de leiomiossarcoma da conjuntiva em um paciente com xeroderma pigmentoso, que também é uma doença rara. Um paciente de 27 anos de idade com xeroderma pigmentoso de olho único queixou-se de uma massa exofítica cobrindo a superfície ocular do olho esquerdo. Ao exame, foi observada uma massa vascular hemorrágica cobrindo toda a superfície ocular do olho esquerdo. Foi realizada uma cirurgia de excisão total dessa massa. O diagnóstico patológico foi compatível com leiomiossarcoma da conjuntiva. O paciente recusou qualquer quimioterapia, radioterapia ou cirurgia adicional. Nenhuma recidiva ou metástase foi observada durante o acompanhamento de 5 anos. Tanto o leiomiossarcoma primário da conjuntiva quanto o xeroderma pigmentoso são doenças muito raras. Massas conjuntivais em pacientes com xeroderma pigmentoso devem ser abordadas com cuidado e deve-se realizar um exame histopatológico. Para o leiomiossarcoma conjuntival, o diagnóstico precoce, uma doença localizada e não disseminada e a ressecção completa proporcionam o melhor prognóstico.

## INTRODUCTION

Leiomyosarcoma is a smooth muscle cell malignancy that is rarely reported as a tumor of the ocular adnexa. Leiomyosarcoma can arise primarily from vascular smooth muscle or ciliary body, occur secondary to radiation therapy, or metastasize from distant sites^(1-3)^. Primary conjunctival leiomyosarcoma has been rarely reported^(4)^. Leiomyosarcoma is clinically difficult to distinguish from other non-pigmented tumors, such as dermoid, lymphoma, and carcinoma. Thus, a histomorphological examination is essential for differential diagnosis.

Xeroderma pigmentosum (XP) is a dermatosis characterized by photo-induced cutaneous-ocular impairment and is related to gene reparation defects. Compared to the normal population, XP patients have an increased risk of developing eye cancer^(5)^. Leiomyosarcoma is not a frequently reported neoplasm in XP patients. Herein, we describe an extremely rare case of a primary conjunctival leiomyosarcoma in a 27-year-old XP patient.

## CASE REPORT

The 27-year-old female patient with XP was admitted and complained of pain and exophytic hemorrhagic mass growing in her left eye. The patient stated that mass excision was made three times in the same region in various centers, but the mass continued to grow rapidly and recurred in the last 15 days.

Her right eye was removed due to an unknown cause 17 years ago, and the pathology and operation reports could not be reached.

**Figure 1:**
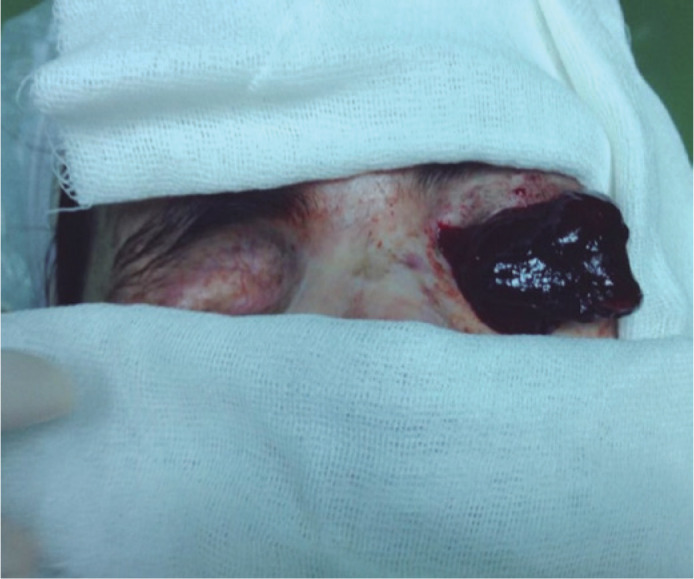
Preoperative lesion appearance: vascular, hemorrhagic, and elevated mass occupying the entire conjunctival and corneal surface.

On ophthalmic examination, the visual acuity was light perception in the left eye. A vascular, hemorrhagic, elevated mass occupying the entire ocular surface and symblepharon in the lower quadrants were observed in the left eye (Figure 1). Excessive hemorrhage-induced globe rupture was suspected. An emergency intervention was planned due to the rapid mass growth, pain, and inability to visualize the anterior segment. Therefore, a detailed preoperative evaluation and imaging could not be performed. Since the tumor was huge and fragile, we performed surgery in a staged manner. Firstly, the tumor apex was excised. After seeing the tumor base, wide total excision, including all base and peripheral tumor tissue, was made.

**Figure 2:**
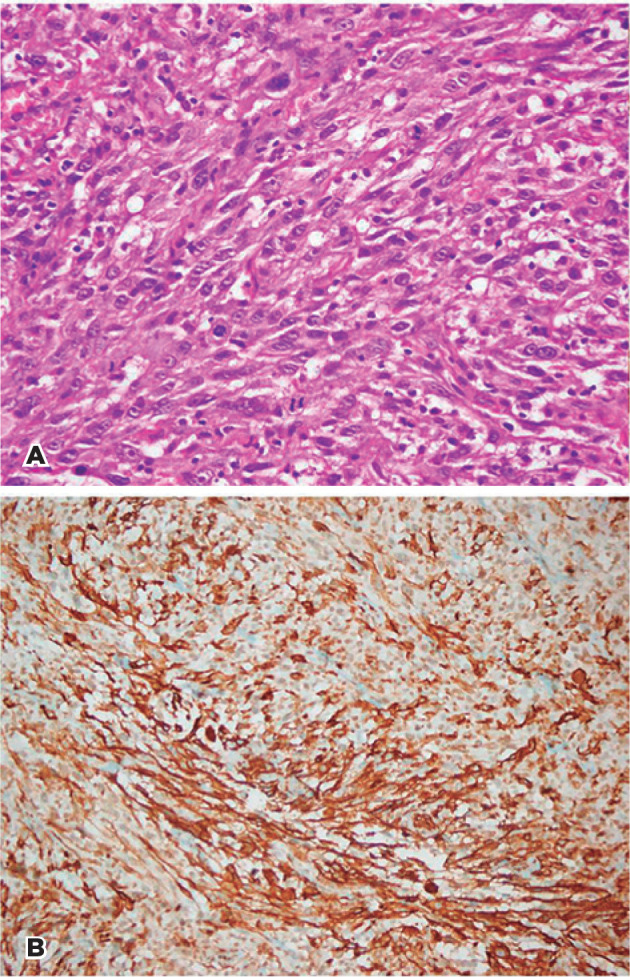
(A). The tumor shows a fascicular pattern, and tu­moral cells have eosinophilic cytoplasm (hematoxylin and eosin (H&E) x400). (B) Tumoral cells are immunopositive for SMA (SMA x200).

Histological examination revealed a malignant tumor composed of spindle-shaped cells, with cellular and nuclear pleomorphism and hyperchromatism combined with eosinophilic cytoplasm. On immunohistochemical staining, the spindle cells showed immunoreactivity for smooth muscle antigen (Figure 2A, B). They were immuno-negative for pancytokeratin markers, S-100, desmin, antigens CD34, and melanocytic marker (HMB45). The tumor cells had a high index of Ki-67 proliferation, which was approximately 70%-82%. Considering morpho­lo­gical and immunohistochemical findings, the pathologi­cal diagnosis was compatible with leiomyosarcoma. Re­section margins were tumor-free.

**Figure 3:**
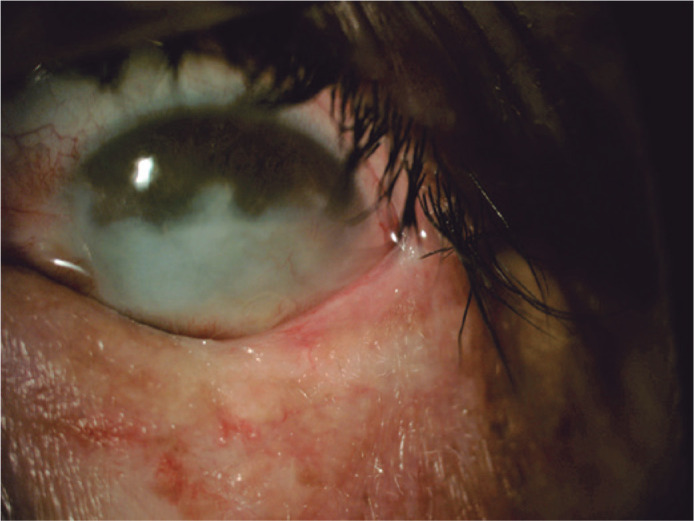
Postoperative ocular surface appearance without recurrence signs.

Since the patient had only one ambulatory eye, she did not accept any further surgical intervention, chemotherapy, or radiotherapy and chose a close follow-up. No evidence of tumor recurrence or systemic metastasis was observed during the 5-year follow-up (Figure 3).

## DISCUSSION

Conjunctival leiomyosarcoma is an extremely rare cancer. Leiomyosarcoma was not detected in the two largest case series in which authors evaluated 2455 and 1643 conjunctival tumors^(6,7)^. A total of 12 cases of conjunctival leiomyosarcoma was reported. De Groot et al. suggested that conjunctival leiomyosarcoma may be caused by small limbal vessels or limbal pluripotent cells because of the limbal localization in most cases^(4)^.

XP is a very rare autosomal-recessive disease seen in 1 per million in the United States and 2.3 per million people in Europe^(8)^. It occurs due to mutations in DNA repair genes, and disease course may differ depending on molecular abnormality and environmental factors. Disease manifestations include freckle-like pigmentation and ocular and skin disorders. The malignancy develops especially in sun-exposed body parts at an earlier age^(8)^.

Ocular involvement in XP usually occurs in areas exposed to ultraviolet radiation, such as the ocular surface and eyelids. It may cause conjunctivitis, photophobia, entropion, exposure keratitis, and pterygium. However, ocular cancer may also develop in these patients^(8)^. Ocular tumors, such as squamous cell carcinoma, basal cell carcinoma, and melanoma, were more frequently reported in XP patients^(8)^. Still, increased ocular leiomyosarcoma incidence is not expected in XP patients. Kaliki et al. evaluated the presence and characteristics of ocular and periocular tumors in 86 XP patients diagnosed with ocular tumors, reporting no leiomyosarcoma cases^(5)^. In the literature, only a single XP patient with suspected conjunctival leiomyosarcoma was reported in 1976, but this case lacked histopathological verification^(9)^. Therefore, it is highly unlikely to detect conjunctival leiomyosarcoma in XP patients. Our case is the first case with histopathological verification in the literature, although the second example of such malignancy since 1976.

Leiomyosarcoma generally has a poor prognosis. However, conjunctival leiomyosarcoma has had a relatively better prognosis due to possible early diagnosis. Without globe invasion, the prognosis may be good, even if the tumor is large^(4)^. The primary treatment for conjunctival leiomyosarcoma is surgical resection, and complete surgical resection is mostly curative. In cases where complete resection is impossible, radiotherapy can be used as adjuvant therapy. In a widespread disease, orbital exenteration is needed^(10)^. Orbital exenteration was performed for conjunctival leiomyosarcoma in the previously reported XP patient^(9)^. Since our case was a single-eyed patient who underwent other-eye enucleation, we had to approach her carefully. The patient underwent complete conjunctival resection due to the clinically malignant lesion appearance. The pathology result was compatible with epithelioid leiomyosarcoma. Additional adjuvant treatment was not applied. No recurrence was identified during the 5-year follow-up.

In XP patients, ocular masses should be carefully interpreted regarding malignancy. Lesion image often does not give an idea about the type of tumor. Histopathological diagnosis is the most valuable tool to determine the clinical approach. We believe that the conjunctival leiomyosarcoma case we presented will contribute to the literature since it is only the second case reported in an XP patient. No recurrence was identified in the 5-year follow-up despite a single resection.
